# Aged garlic extract and S-allylcysteine prevent apoptotic cell death in a chemical hypoxia model

**DOI:** 10.1186/s40659-016-0067-6

**Published:** 2016-02-01

**Authors:** Marisol Orozco-Ibarra, Jorge Muñoz-Sánchez, Martín E. Zavala-Medina, Benjamín Pineda, Roxana Magaña-Maldonado, Edgar Vázquez-Contreras, Perla D. Maldonado, José Pedraza-Chaverri, María Elena Chánez-Cárdenas

**Affiliations:** Laboratorio de Neurobiología Molecular y Celular, Instituto Nacional de Neurología y Neurocirugía Manuel Velasco Suárez, Delegación Tlalpan, 14269 Mexico, D.F., Mexico; Laboratorio de Patología Vascular Cerebral, Instituto Nacional de Neurología y Neurocirugía Manuel Velasco Suárez, Av. Insurgentes Sur # 3877, Colonia La Fama, Delegación Tlalpan, 14269 Mexico D.F., Mexico; Laboratorio de Neuroinmunología y Neuro-oncología, Instituto Nacional de Neurología y Neurocirugía Manuel Velasco Suárez, Delegación Tlalpan, 14269 Mexico D.F., Mexico; Departamento de Ciencias Naturales, CNI, Universidad Autónoma Metropolitana Cuajimalpa, Av. Vasco de Quiroga 4871: Col. Santa Fe, Delegación Cuajimalpa de Morelos, 05348 Mexico D.F., Mexico; Departamento de Biología, Facultad de Química, Universidad Nacional Autónoma de México, Ciudad Universitaria, 04510 Mexico D.F., Mexico

**Keywords:** Aged garlic extract, Chemical hypoxia, Cobalt chloride, Hypoxia-inducible factor 1, PC12 cells, S-allylcysteine

## Abstract

**Background:**

Aged garlic extract (AGE) and its main constituent S-allylcysteine (SAC) are natural antioxidants with protective effects against cerebral ischemia or cancer, events that involve hypoxia stress. Cobalt chloride (CoCl_2_) has been used to mimic hypoxic conditions through the stabilization of the α subunit of hypoxia inducible factor (HIF-1α) and up-regulation of HIF-1α-dependent genes as well as activation of hypoxic conditions such as reactive oxygen species (ROS) generation, loss of mitochondrial membrane potential and apoptosis. The present study was designed to assess the effect of AGE and SAC on the CoCl_2_-chemical hypoxia model in PC12 cells.

**Results:**

We found that CoCl_2_ induced the stabilization of HIF-1α and its nuclear localization. CoCl_2_ produced ROS and apoptotic cell death that depended on hypoxia extent. The treatment with AGE and SAC decreased ROS and protected against CoCl_2_-induced apoptotic cell death which depended on the CoCl_2_ concentration and incubation time. SAC or AGE decreased the number of cells in the early and late stages of apoptosis. Interestingly, this protective effect was associated with attenuation in HIF-1α stabilization, activity not previously reported for AGE and SAC.

**Conclusions:**

Obtained results show that AGE and SAC decreased apoptotic CoCl_2_-induced cell death. This protection occurs by affecting the activity of HIF-1α and supports the use of these natural compounds as a therapeutic alternative for hypoxic conditions.

**Electronic supplementary material:**

The online version of this article (doi:10.1186/s40659-016-0067-6) contains supplementary material, which is available to authorized users.

## Background

Hypoxia is a component of physiological events and multiple pathophysiological conditions, including neonatal hypoxia, cerebral ischemia, peripheral vascular disease, myocardial infarction, coronary heart disease and cancer [1, 2]. Hypoxic stress to cells alters gene expression regulation, leading to subsequent recovery processes or cell death. Hypoxia-inducible factor 1 (HIF-1) is the master regulator of oxygen homeostasis, and adaptive cellular responses to reduced oxygen availability are primarily regulated by the stabilization/degradation ratio of the α subunit of the transcription factor HIF-1 [3–5].

Cobalt chloride (CoCl_2_) has been widely used to imitate hypoxic conditions both in vivo [6, 7] and in vitro [8, 9]. CoCl_2_ mimics several aspects of the hypoxic response, such as increasing and stabilizing HIF-1α protein through inhibition of Prolyl Hydroxylases (PHDs) activity and producing reactive oxygen species (ROS), which leads to cell damage, decreased cell viability and apoptosis [9–11]. Previous studies have clearly shown that CoCl_2_-induced cell damage is associated with an increase in ROS that subsequently induces apoptosis. Apoptotic morphology, DNA fragmentation, activation of caspases -3 and -9, loss of mitochondrial membrane potential, cytochrome c release, upregulation of Bax and down-regulation of Bcl2, [8, 9, 12–16] as well as p38MAPK activation [13] has been observed as result of CoCl_2_ induced damage.

The current study assessed the mechanism of action and protective effect of two antioxidant garlic derivatives, aged garlic extract (AGE) and S-allylcysteine (SAC), on hypoxia-induced damage.

AGE is the result of prolonged (20 months) garlic extraction in 20 % ethanol. This aging process converts unstable and odorous compounds into odorless and stable forms [17, 18]. The most abundant organosulfur compound in AGE is SAC (0.6 mg/g product); however, other AGE compounds, such as S-allylmercaptocysteine, alliin, Nα-(1-deoxy-D-fructos-1-yl)-l-arginine and tetrahydro-beta-carbolines, have been shown to inhibit oxidizing events and to scavenge free radicals and oxidant species such as hydroxyl radicals (^•^OH), superoxide anions (O_2_^•-^) and hydrogen peroxide (H_2_O_2_). The evidence shows that AGE can ameliorate the oxidative damage implicated in aging and a variety of diseases, including cardiovascular alterations, cancer, stroke, Alzheimer’s disease, and other age-related degenerative conditions (reviewed in [19]). The protective effect of AGE in different models has been associated with its antioxidant properties [20–22], reviewed in [23].

SAC is the best characterized AGE compound. It is formed by γ-glutamyl-S-allylcysteine catabolism and has been used to standardize commercial AGE [24]. Previous studies have shown that SAC scavenges O_2_^•^^−^ [25], H_2_O_2_, ^•^OH, peroxynitrite anions, hypochlorous acid, singlet oxygen and peroxyl radicals [25, 26]. It also prevents H_2_O_2_-induced peroxidation and H_2_O_2_-induced activation of NFκB. SAC administration increases glutathione levels as well as catalase and glutathione peroxidase activities [19].

CoCl_2_-treated PC12 cells have been used to study the mechanisms underlying cell death due to hypoxia/ischemia conditions because they are particularly sensitive to hypoxic changes and reproduce apoptotic cell death [8, 9, 12–14]. We investigated the effect of AGE and SAC on cell death to determine the mechanisms behind CoCl_2_-induced injuries in PC12 cells. Our results showed that the protective effect of SAC and AGE was due to the preservation of cell viability and a decrease in cell death, particularly CoCl_2_-induced apoptosis. Our observations suggest that this increased cell survival occurs through the attenuation of HIF-1α stabilization and binding activity via the direct antioxidant effects of AGE and SAC.

## Results

### CoCl_2_ affects cell viability in a concentration- and time-dependent manner

**Fig. 1:**
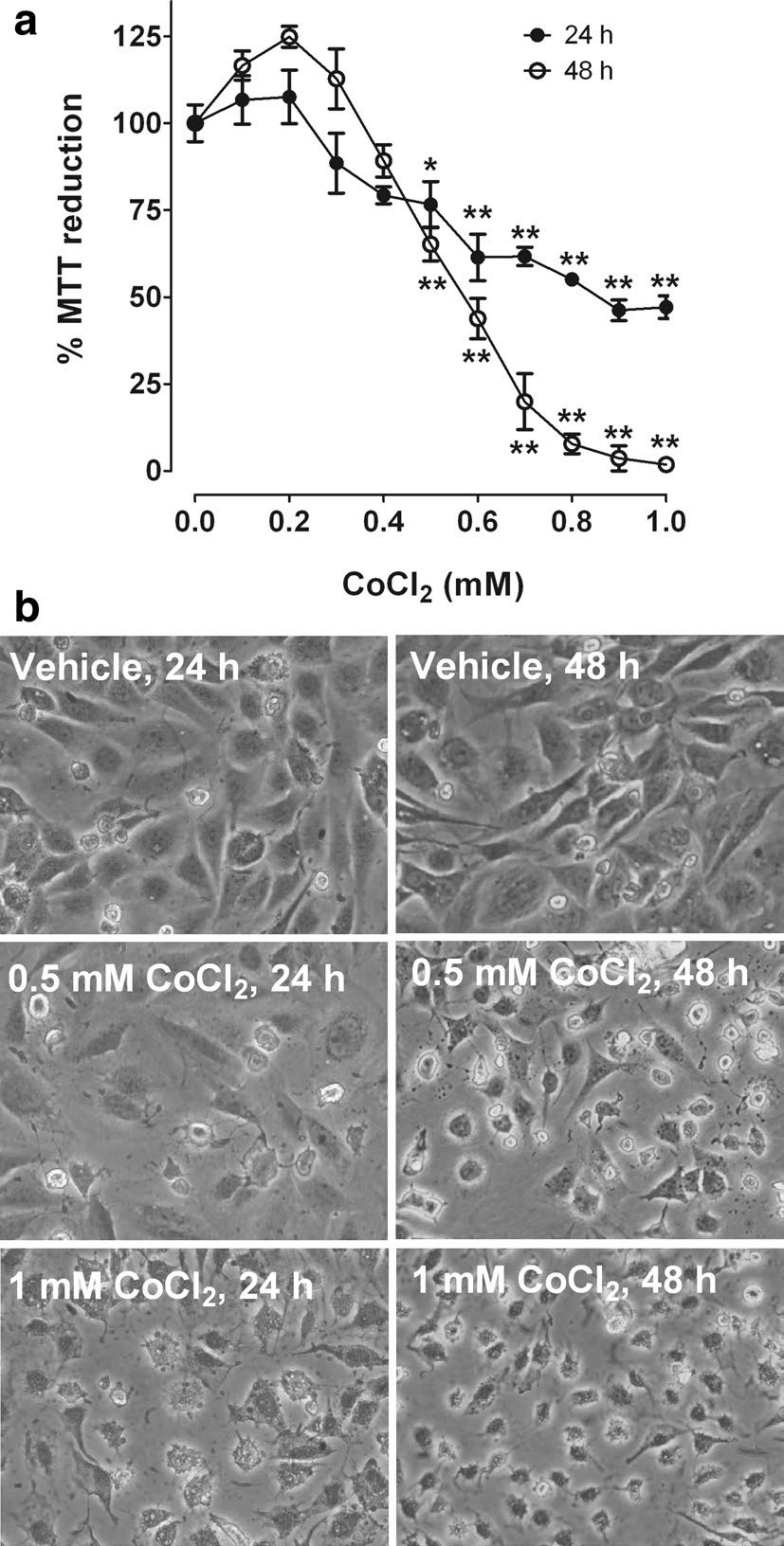
Effect of CoCl_2_ on cell viability. Cells were incubated for 24 or 48 h at increasing CoCl_2_ concentrations (0.1–1.0 mM). After incubation, (**a**) the MTT reduction was determined, and (**b**) representative phase-contrast micrographs showing the effect of CoCl_2_ on cell morphology were taken. *Data* are shown as the mean ± S.E.M, n = 3–4. One-way ANOVA followed by Dunnett’s test. *p < 0.05 and **p < 0.01 vs. 0 mM (control)

Concentration- and time-dependent changes in MTT reduction were used to assess CoCl_2-_induced effects. Cells were incubated for 24 or 48 h at increasing concentrations of CoCl_2_. Concentration- and time-dependent decreases in cell viability were observed. A significant reduction was observed at 0.5 mM CoCl_2_ for both time periods tested (Fig. 1a). The lowest cell viability percentage was observed after 24-h incubation with 1.0 mM CoCl_2_ (50 %). At 48 h, a 50 % MTT reduction was observed after exposure between 0.4 and 0.6 mM CoCl_2_. Representative bright field micrographs of cells at 0 (vehicle), 0.5 and 1.0 mM CoCl_2_ at 24 or 48 h are shown in Fig. 1b. Time-dependent cell shrinkage and irregular shapes were found in cells treated with 0.5 and 1.0 mM CoCl_2_. These data are consistent with the MTT reduction results.

### CoCl_2_ stabilizes HIF-1α and increases binding to hypoxia response elements (HRE) sequence

**Fig. 2:**
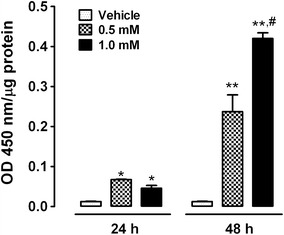
Effect of CoCl_2_ on HIF-1α stabilization and binding activity. Cells were incubated for 24 or 48 h with 0.5 or 1.0 mM CoCl_2_. Binding of nuclear HIF-1α to HRE sequences was determined using an ELISA. *Data* are shown as the mean ± S.E.M, n = 3. Two-way ANOVA followed by Bonferroni comparisons. *p < 0.05 and **p < 0.001 vs. vehicle at the same time point, ^#^p < 0.001 vs. 0.5 mM CoCl_2_ at the same time point

The binding of stabilized HIF-1α was determined using ELISA (Fig. 2). The binding of nuclear HIF-1α to HRE sequence was increased by approximately 6- and 4-fold after a 24-h exposure to 0.5 and 1.0 mM CoCl_2_, respectively. CoCl_2_ incubation for 48 h induced further increases of 19- and 35-fold after exposure to 0.5 and 1.0 mM CoCl_2_, respectively. No signal was detected in cells incubated with 0.15 mM CoCl_2_ for 30 min or 1 h (data not shown). Based on these results and the MTT data (Fig. 1), we used exposure to 0.5 and 1.0 mM CoCl_2_ for 24 or 48 h for subsequent experiments.

### SAC and AGE prevent CoCl_2_-induced toxicity

**Fig. 3:**
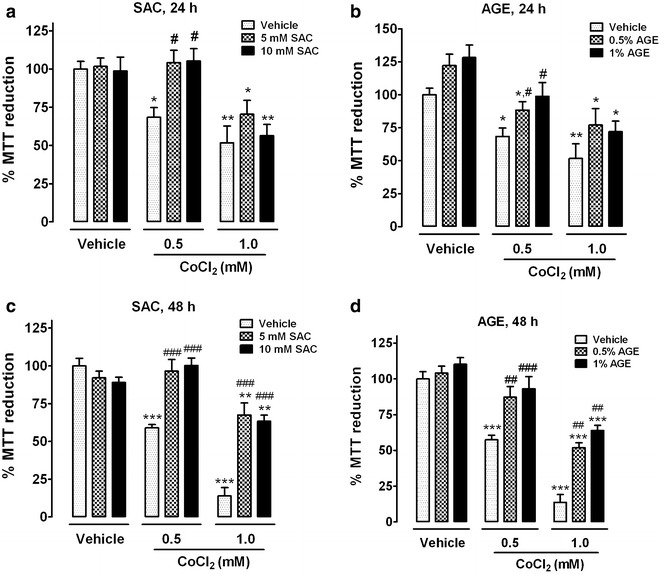
Effect of SAC and AGE on CoCl_2_-induced toxicity in PC12 cells. Cells were co-incubated with CoCl_2_ and either SAC or AGE for 24 (**a** and **b**) or 48 h (**c** and **d**). *Data* are shown as the mean ± S.E.M. n = 4. Two-way ANOVA followed by Bonferroni comparisons. *p < 0.05, **p < 0.01 and ***p < 0.001 vs. vehicle and ^#^p < 0.05, ^##^p < 0.01 and ^###^p < 0.001 vs. the same concentration of CoCl_2_

To determine the effect of SAC and AGE on CoCl_2_-induced toxicity, cells were co-incubated with SAC or AGE and CoCl_2_ for 24 or 48 h as stated in the experimental design. The level of MTT reduction was determined. Concentrations of 5 or 10 mM SAC and 0.5 or 1.0 % AGE were chosen based on previous in vitro reports (SAC: [27, 28]; AGE: [29, 30]) and toxicity experiments using SAC (0–20 mM) or AGE (0–1 %) for 24 and 48 h (data not shown). After 24 h, 0.5 mM CoCl_2_ reduced cell viability to 60 %, and co-incubation with SAC (5 or 10 mM) completely restored cell viability (Fig. 3a). Similar results were obtained with AGE, including a partial increase in cell viability after treatment with 0.5 % AGE and almost complete prevention with 1.0 % AGE after cells were incubated with 0.5 mM CoCl_2_ (Fig. 3b). Neither SAC (Fig. 3a) nor AGE (Fig. 3b) exhibited a significant protective effect on the toxicity induced by 1.0 mM CoCl_2_. The toxicity induced by 0.5 mM or 1.0 mM CoCl_2_ for 48 h was clearly prevented by co-incubation with either SAC (Fig. 3c) or AGE (Fig. 3d). Based on these results, subsequent experiments were conducted using 10 mM SAC and 1 % AGE for 48 h.

### SAC and AGE prevent cell death induced by CoCl_2_

**Fig. 4:**
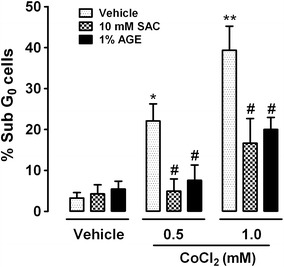
Effect of SAC or AGE co-incubation with CoCl_2_ on the Sub-G_0_ peak. Cells were co-incubated with 10 mM SAC or 1 % AGE and 0.5 or 1.0 mM CoCl_2_ for 48 h. Sub-G_0_ data were obtained using flow cytometry with cells incubated with PI. *Data* are shown as the mean ± S.E.M. n = 4–5. Two-way ANOVA followed by Bonferroni comparisons.*p < 0.01 and **p < 0.001 vs. control cells (vehicle), ^#^p < 0.01 vs. the same concentration of CoCl_2_

To further investigate the effect of SAC and AGE on CoCl_2_-induced cell death, we monitored the cell cycle profile using fluorescence-activated cell sorting (FACS) analysis (Fig. 4). The fraction of cells in the Sub-G_0_ phase increased from 3 to 22 % after exposure to 0.5 mM CoCl_2_ for 48 h and 39 % after exposure to 1.0 mM CoCl_2_ (compared to vehicle). Both SAC and AGE prevented this increase. Co-incubation with SAC and AGE reduced the 0.5 mM CoCl_2_-induced cell death to 5 and 8 %, respectively. Cells exposed to 1.0 mM CoCl_2_ and SAC or AGE showed a decrease in cell death from 39 to 17 and 20 %, respectively.

### SAC and AGE prevent CoCl_2_-induced apoptosis

**Fig. 5:**
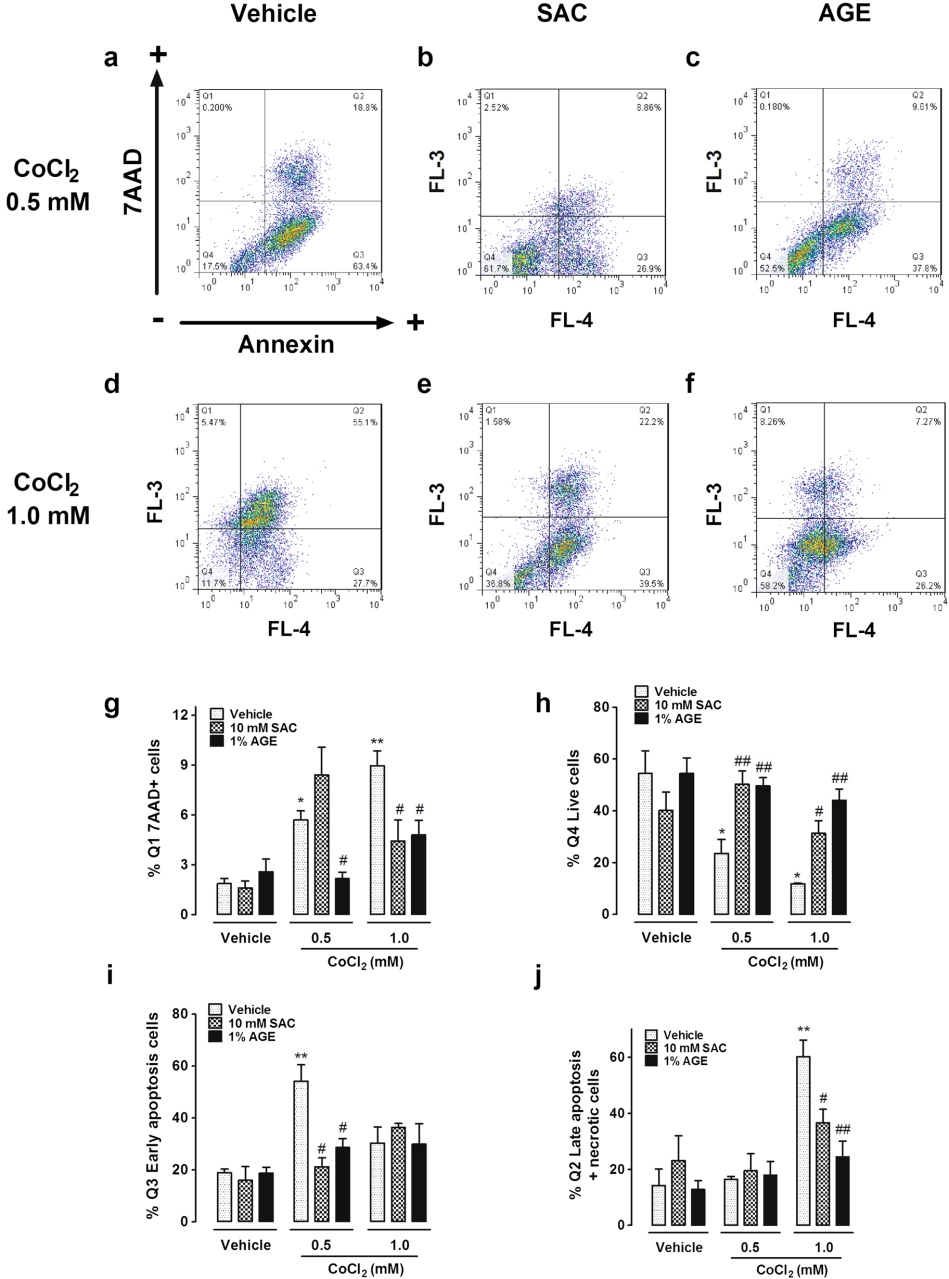
Protective effect of SAC and AGE on CoCl_2_-induced apoptosis. *Upper panel*: Representative figures of Annexin and 7-AAD double staining assay using flow cytometry after 48-h incubation with CoCl_2_ and SAC or AGE (**a**–**f**). Percentage of 7-AAD + (**g**), live Annexin-/7AAD- (**h**), early apoptotic Annexin +/7AAD- (**i**) and late apoptotic plus necrotic cells Annexin +/7AAD + (**j**). *Data* are shown as the mean ± S.E.M. n = 6. Two-way ANOVA followed by Bonferroni comparisons. *p < 0.05, **p < 0.001 vs. control cells (vehicle), ^#^p < 0.05, ^##^p < 0.01 vs. the same concentration of CoCl_2_

The Annexin V/7-AAD staining in Fig. 5 shows the effect of CoCl_2_ and SAC or AGE on cell death. Representative figures are shown in Fig. 5 (a–f). The analysis of six independent experiments is shown in Fig. 5 (g–j). In agreement with the MTT reduction and Sub-G_0_ peak results, SAC and AGE prevented CoCl_2_- induced cell death. The known apoptosis inducer in PC12 cells staurosporine (200 nM) was used as a positive control (Additional file 1: Figure S1). The percentage of live cells at 0.5 mM CoCl_2_ was 22 %, and co-incubation with SAC or AGE increased cell viability to 50 %. Co-incubation of cells with 1.0 mM CoCl_2_ and SAC or AGE prevented cell death and increased the percentage of live cells from 8 to 30 and 40 %, respectively (Fig. 5h). Single 7-AAD + cells were less than 10 % for both CoCl_2_ concentrations (Fig. 5g). Early apoptotic cells (single Annexin +) increased from 15 % to approximately 50 % after exposure to 0.5 mM CoCl_2_. This increase was prevented by co-incubation with SAC (to 20 %) or AGE (to 25 %) (Fig. 5i). In addition, 1.0 mM CoCl_2_ induced an increase in Annexin +/7-AAD + cells from 15 % to approximately 60 %, and both SAC and AGE attenuated this effect to 35 and 18 %, respectively (Fig. 5 j).

### SAC and AGE decrease CoCl_2_-induced ROS generation

**Fig. 6:**
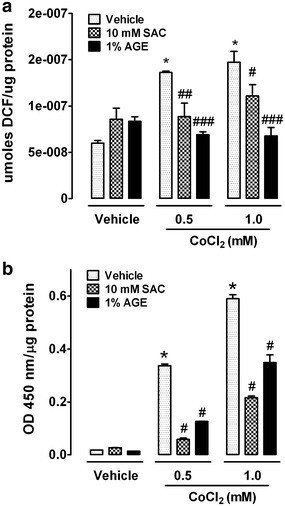
SAC and AGE attenuates CoCl_2_-induced overproduction of ROS and HIF-1α stabilization and binding activity. **a** Cells were incubated with CoCl_2_ and SAC or AGE to determine intracellular ROS levels. *Data* are shown as the mean ± S.E.M. n = 6. *p < 0.001 vs. vehicle. ^#^p < 0.05, ^##^p < 0.01 and ^###^p < 0.001 vs. the same concentration of CoCl_2_. **b** Binding of stabilized HIF-1α to HRE sequences was determined using an ELISA. *Data* are shown as the mean ± S.E.M. n = 3. Two-way ANOVA followed by Bonferroni comparisons. *p < 0.001 vs. vehicle; ^#^p < 0.001 vs. the same concentration of CoCl_2_

In addition to the increase in HIF-1α protein stabilization, CoCl_2_ mimics other hypoxia responses, including ROS generation. Figure 6a shows the increase in the 2’, 7’dichlorofluorescein (DCF)-derived fluorescence of cells exposed for 1 h to 0.5 and 1.0 mM CoCl_2_ (vehicle bars). Co-incubation with 10 mM SAC or 1 % AGE decreased the ROS generated from exposure to both CoCl_2_ concentrations.

### SAC and AGE decrease CoCl_2_-induced HIF-1α stabilization and binding to HRE sequence

The effect of SAC and AGE on nuclear HIF-1α stabilization and binding to HRE sequences was tested using an ELISA. A significant increase in the HIF-1α signal was observed at 0.5 and 1.0 mM CoCl_2_ (20- and 35-fold, respectively), and SAC and AGE prevented the increase in HIF-1α. Exposure to 0.5 mM CoCl_2_ decreased HIF-1α binding activity from 20-fold to 3-fold with SAC and 7-fold with AGE. At 1.0 mM CoCl_2_, HIF-1α activity decreased from 35-fold to 13-fold and 19-fold with SAC or AGE, respectively (Fig. 6b).

## Discussion

The cellular injury caused by hypoxia is involved in pathological events, such as cerebral ischemia, neonatal hypoxia and cancer. Thus, it is relevant to explore the mechanisms underlying the potential protective effects of compounds through hypoxia models.

The chemical hypoxia model induced by CoCl_2_ exposure in PC12 cells is a useful in vitro model to elucidate the mechanisms behind hypoxia damage and test novel compounds because it reproduces many hypoxic conditions, including ROS generation, mitochondrial membrane potential changes, induction of apoptosis [12–16] and HIF-1α-regulated transcriptional responses [8, 9]. In this study, we observed that exposure to 0.5 and 1.0 mM CoCl_2_ for 24 or 48 h induced the stabilization and translocation of HIF-1α to the nucleus, confirming that CoCl_2_ can mimic hypoxia in PC12 cells [8, 15].

This study was designed to determine the effect of two garlic-derived antioxidants, AGE and SAC, in PC12 cells subjected to chemical hypoxia induced by CoCl_2_. We observed that co-incubation of AGE or SAC with CoCl_2_ exerted a protective effect against the CoCl_2_-induced cell viability decrease. In the case of 1.0 mM CoCl_2_, it was clearly evident only after longer periods of exposure (48 h). Cell function was almost completely recovered for SAC or AGE co-incubation with 0.5 mM CoCl_2_ at both tested times. In contrast, 1.0 mM CoCl_2_ had a pronounced toxicity effect that results in an apparent lack of protection at both SAC and AGE concentrations tested at 24 h (Fig. 3a, b). Interestingly, a protective effect exerted by SAC and AGE was observed at 48 h, with a significant recovery of the cell viability at 1.0 mM CoCl_2_ (Fig. 3c, d). Noteworthy, about the same percentage of viable cells observed at 24 h with SAC and AGE treatment was maintained at 48 h. It suggests that both compounds retain cell viability along time. The marked decrease of cell viability in vehicle treated cells (48 h compared to 24 h) evidences this protective effect. SAC and AGE did not show a dose dependent effect, probably because the recovery of cell function at 5 mM SAC or 0.5 % AGE is the maximum recovery we could obtain for the 1.0 mM CoCl_2_-induced damage in PC12 cells. We suggest that protective mechanisms of both SAC and AGE may depend on the severity of the stimulus, which could trigger different molecular responses, such as the differences observed in HIF-1α activity at both CoCl_2_ concentrations (Fig. 2).

The decrease in Sub-G_0_ values after treatment with SAC or AGE confirmed this protection. This technique allows quantification of the number of cells through DNA fragmentation. To differentiate early apoptotic cells, we performed flow cytometry with Annexin V/7-AAD double staining. Sub-G_0_ results were corroborated by the Annexin V-7AAD assay, and a significant preservation of the number of live cells was observed after AGE or SAC treatment. Although this assay does not distinguish between late apoptotic and necrotic cells since both of them are Annexin and 7-AAD positive (Q2: Annexin +/7-AAD +), previous characterization of CoCl_2_-induced cell death strongly suggest that the cells in Q2 are mainly apoptotic. In addition, we used another well-known apoptotic inducer in PC12 cells (Additional file 1: Figure S1). As expected, and in accordance to CoCl_2_ effect, cells are mainly distributed in Q2 and Q3 in response to staurosporine 200 nM. Since we strictly cannot differentiate necrotic cells, we labeled Q2 as late apoptotic and necrotic cells. Observed cell death depended on the CoCl_2_ concentration and incubation time; furthermore, SAC or AGE decreased the number of cells in early apoptosis as well as in late stages of apoptosis (plus possible necrotic cells).

AGE and SAC attenuated HIF-1α stabilization and binding to HRE sequences, suggesting a direct effect on the transcriptional activity of HIF-1α. Several reports suggest that ROS induces HIF-1α protein stabilization [31, 32]. It has been reported that ROS activates the HIF-1α promoter via a NFκB site, suggesting that these factors are important in disorders that show increased levels of ROS [33]; in addition, the effects of ROS on HIF-1α can also be affected by the degree of hypoxia, the form and intracellular location of ROS produced during hypoxia and the molecular microenvironment of the cell [34]. Under hypoxic conditions, mitochondrial complex III may produce ROS, and the presence of high ROS concentrations generated from the mitochondria has been shown to stabilize HIF-1α [35–38]. However, it has been suggested that mitochondrial-independent mechanisms are primarily responsible for CoCl_2_-induced ROS generation and the activation of HIF-1α [10, 11]. However, the generation of ROS could also be due to NADPH oxidases in the cytosol. Regardless, ROS influence HIF-1α activity. We observed that a decrease in activated HIF-1α correlates with a decrease in CoCl_2_-induced ROS after SAC or AGE treatment. There are several hypotheses that can explain the stabilization of HIF-1α expression by ROS: 1) Fe^2+^ oxidation by H_2_O_2_ affects PHDs activity; 2) prevention of Fe^3+^ reduction by ascorbate; 3) prevention of ascorbate from binding to PHDs; 4) or a ROS-induced change in O_2_ availability that affects HIF binding to PHDs [34]. The concentrations of CoCl_2_ used in our study have been shown to induce oxidant stress in cells and oxidize intracellular ascorbate [39], confirming that ROS affects HIF-1α stability.

Thus, SAC and AGE prevent HIF-1α activation due to their antioxidant nature, which leads to a decrease in apoptotic cell death. The antioxidant effect of SAC and AGE on HIF-1α activity has not been described to date. A reduction in HIF-1α levels has also resulted in protection in other models [14, 39–41]. The reduction of cell death by blocking HIF-1α stabilization may be due to the inability of HIF-1α to induce the transcription of proapoptotic members of the Bcl2 BH3-only family that induce oxidative death, such as BNIP3, PUMA and NOXA [42–44]. Hence, the prevention of apoptotic cell death by SAC and AGE can be explained by the abrogation of HIF-mediated transactivation of BH3-only proteins [42].

In summary, we observed a protective effect of AGE and SAC on CoCl_2_ toxicity after CoCl_2_ exposure. The cell viability was preserved by AGE or SAC co-incubation. Flow cytometry showed that both garlic-derived antioxidants decreased the number of dead cells and a decrease in early and late apoptotic cell death after AGE or SAC treatment, depending on the CoCl_2_ concentration and incubation time used. Both garlic derivatives decreased HIF-1α stabilization and nuclear translocation, suggesting that their antioxidant role directly affects the transcriptional activity of HIF-1α and subsequently blocks the transcription of prodeath HIF-1α regulated genes.

## Conclusions

HIF modulation is an attractive strategy for the therapeutic intervention of pathological conditions affected by hypoxia and for the study of hypoxic mechanisms; however, fundamental studies are required before testing in clinical trials. We suggest that the use of SAC or AGE is a plausible strategy to minimize the prodeath effects of HIF-1α and the consequent cell death associated with hypoxia and oxidative stress conditions. However, further research is necessary to elucidate the exact signaling pathways involved. The mechanisms behind apoptosis and its relationship with HIF-1α activity can be used to translate the full potential of AGE and SAC into a preventive strategy to counteract hypoxic consequences.

## Methods

### Materials

All reagents were analytical grade and commercially available. AGE Kyolic^®^ was obtained from Wakunaga of America Co. (Ltd, Mission Viejo, CA, USA). This garlic extract complies with the specifications established in the US Pharmacopeia/National Formulary [24].

### Synthesis of S-allylcysteine (SAC)

SAC was synthesized by the reaction of l-cysteine with allyl bromide and purified by recrystallization from an ethanol–water solution. The final product was compared with a SAC standard using thin layer chromatography, high performance liquid chromatography, ^1^H nuclear magnetic resonance (NMR), infra-red and electronic ionization-mass spectroscopy (EI-MS). A detailed procedure for SAC synthesis, purification and identification of the final chemical compound are reported in [26]. The melting point of the SAC standard is 220–222 °C, and the melting point of the SAC used in this study was 218–219 °C. The analysis of the final product by high performance liquid chromatography showed one peak with a retention time of 12.38 min.

### Cell culture

PC12 cells were obtained from the American Type Culture Collection (Rockville, MD, USA). Cells were routinely cultured in Dulbecco’s modified Eagle’s Medium (DMEM) with 7.5 % heat-inactivated horse serum and 7.5 % fetal bovine serum at 37 °C in a humidified atmosphere of 5 % CO_2_/95 % air [14]. Cells were seeded at a density of 7.5 × 10^4^ cells/cm^2^. For experiments, confluent cultures were maintained in DMEM free of serum.

### Experimental design

Cells were incubated for 24 or 48 h at 37 °C in one of the following conditions: vehicle (DMEM free of serum); SAC (5 or 10 mM); AGE (0.5 or 1.0 %); CoCl_2_ (0.5 or 1.0 mM); CoCl_2_ 0.5 mM plus SAC (5 or 10 mM); CoCl_2_ 1.0 mM plus SAC (5 or 10 mM); CoCl_2_ 0.5 mM plus AGE (0.5 or 1.0 %); or CoCl_2_ 1.0 mM plus AGE (0.5 or 1.0 %). Cells were harvested and used to evaluate HIF-1α activation, cell viability and Sub-G_0_ levels using an enzyme-linked immunosorbent assay (ELISA), 3-(4,5-dimethylthiazol-2-yl)-2,5-diphenyltetrazolium bromide (MTT) reduction assay and Annexin V/7-aminoactinomycin D (7-AAD) double staining with flow cytometry, respectively. Intracellular ROS levels were determined using 2’,7’dichlorofluorescein diacetate (DCFH-DA) in cells incubated for 1 h under the aforementioned conditions.

### Cell viability assay

Cell viability was assessed using a MTT reduction assay as previously reported [14]. MTT (0.5 mg ml^−1^) was added to each well, and cells were incubated at 37 °C for 1.5 h. The formazan blue product was spectroscopically quantified at 570 nm. Data are expressed as the percentage of MTT reduction compared to control wells. The data were confirmed by bright field micrographs.

### HIF-1α activity assay

Cell nuclear fractions were obtained using a NE-PER^®^ Nuclear and Cytoplasmic Extraction Reagents kit, (PIERCE, Thermo Scientific, Rockford, IL, USA), and the protein concentration was determined. Stabilized HIF-1α bound to the HRE sequence was determined using an ELISA-HIF-1α activity Transcription Factor Assay Kit (Cayman Chemical Co. Ann Arbor, MI, USA) according to the manufacturer’s instructions. The results are expressed as OD 450 nm/µg protein.

### Flow cytometry

Based on the methods of [45], FACS analysis was used to determine the Sub-G_0_ peak and the level of apoptosis after staining with propidium iodide (PI) or Annexin V and 7-AAD, respectively. A total of 10,000 events were evaluated, and data were collected on a FACSCalibur instrument (BD Biosciences, Franklin Lakes, NJ, USA). Data analysis was conducted using Cell QuestPro and Flow Jover 7.6.1 software.

### Reactive oxygen species measurement

Intracellular ROS levels were determined by the oxidative conversion of DCFH-DA to DCF. PC12 cells were cultured in 6-well plates. Cells were incubated for 1 h using the previously described treatment conditions. Cells were washed twice with PBS, and the 10 µM DCFH-DA in serum-free medium was added. Next, cells were incubated for 30 min at 37 °C in the dark. Cells were washed twice with PBS, lysed in phosphate buffer (50 mM pH 7.4, 1 % v/v Triton X-100) and centrifuged for 20 min at 12,000*g*. The fluorescence intensity was determined at excitation/emission wavelengths of 488 nm/515 nm. Micromoles of DCF were determined with a DCF calibration curve.

### Statistical analyses

Data are expressed as the mean ± S.E.M. and were analyzed using Prism 5 software (GraphPad, San Diego, CA, USA), applying analysis of variance (ANOVA) followed by the Bonferroni Multiple Comparison test or Dunnett’s test, as appropriate. A value of p < 0.05 was considered significant.

## Additional file

10.1186/s40659-016-0067-6 Positive control for PC12 cells apoptosis induced by staurosporine. PC12 cells were incubated in the same conditions described in Methods. Representative figure of flow cytometry for Annexin and PI double staining assay after 24 h incubation with staurosporine 200 nM (a). Graph shows results from three different experiments. Q1, PI single positive cells; Q2, Annexin +/IP + (late apoptotic and necrotic cells); Q3, Annexin +/IP- early apoptotic cells; Q4, Annexin-/IP- live cells (b). Data are shown as the mean ± S.E.M. n = 3.

## Abbreviations

DCFH-DA: 2’,7’dichlorofluorescein diacetate
DCF: 2’, 7’dichlorofluorescein
MTT: 3-(4,5-dimethylthiazol-2-yl)-2,5-diphenyltetrazolium bromide
7-AAD: 7-Aminoactinomycin D
AGE: aged garlic extract
ANOVA: analysis of variance
CoCl_2_: cobalt chloride
DMEM: Dulbecco’s modified eagle’s medium
ELISA: enzyme-linked immunosorbent assay
H_2_O_2_: hydrogen peroxide
HIF-1: hypoxia inducible factor
HRE: hypoxia response elements
O_2_: oxygen
PI: propidium iodide
ROS: reactive oxygen species
SAC: S-allylcysteine
