# Supplementary figures and images for: Aged garlic extract and S-allylcysteine prevent apoptotic cell death in a chemical hypoxia model
Source: Biol Res. 2016 Feb 1;49:7. doi: 10.1186/s40659-016-0067-6 (PMC4736283; doi:10.1186/s40659-016-0067-6)

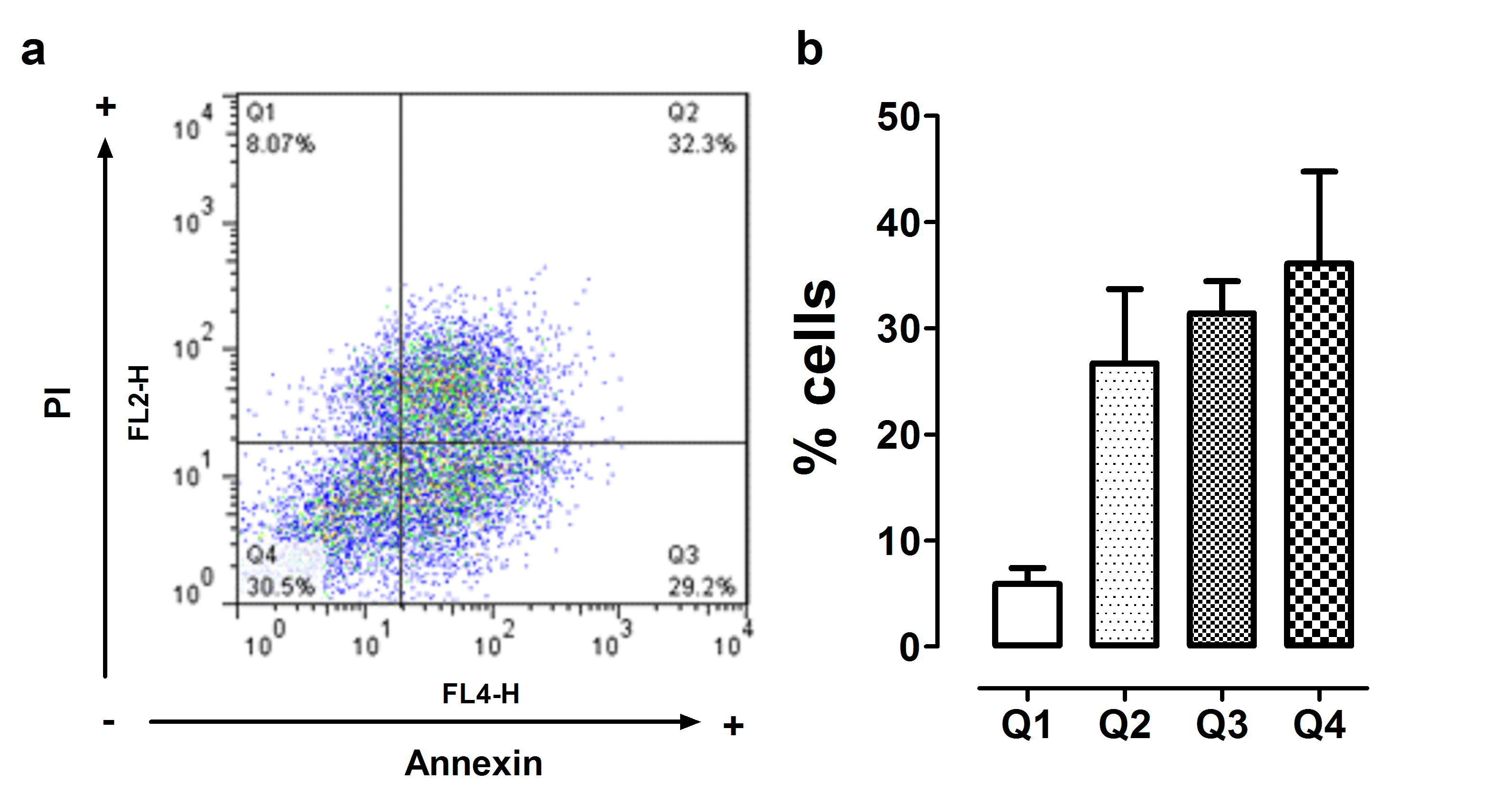

Supplement: Supplementary file 1 — 10.1186/s40659-016-0067-6 Positive control for PC12 cells apoptosis induced by staurosporine. PC12 cells were incubated in the same conditions described in Methods. Representative figure of flow cytometry for Annexin and PI double staining assay after 24 h incubation with staurosporine 200 nM (a). Graph shows results from three different experiments. Q1, PI single positive cells; Q2, Annexin +/IP + (late apoptotic and necrotic cells); Q3, Annexin +/IP- early apoptotic cells; Q4, Annexin-/IP- live cells (b). Data are shown as the mean ± S.E.M. n = 3. [file 40659_2016_67_MOESM1_ESM.tif]
